# Induction of TLR-2 and TLR-5 Expression by *Helicobacter pylori* Switches *cag*PAI-Dependent Signalling Leading to the Secretion of IL-8 and TNF-α

**DOI:** 10.1371/journal.pone.0019614

**Published:** 2011-05-09

**Authors:** Suneesh Kumar Pachathundikandi, Sabine Brandt, Joseph Madassery, Steffen Backert

**Affiliations:** 1 Institute for Medical Microbiology, Otto von Guericke University, Magdeburg, Germany; 2 Department of Biotechnology, University of Calicut, Calicut University (PO), Kerala, India; Veterans Affairs Medical Center (111D), United States of America

## Abstract

*Helicobacter pylori* is the causative agent for developing gastritis, gastric ulcer, and even gastric cancer. Virulent strains carry the *cag* pathogenicity island (*cag*PAI) encoding a type-IV secretion system (T4SS) for injecting the CagA protein. However, mechanisms of sensing this pathogen through Toll-like receptors (TLRs) and downstream signalling pathways in the development of different pathologies are widely unclear. Here, we explored the involvement of TLR-2 and TLR-5 in THP-1 cells and HEK293 cell lines (stably transfected with TLR-2 or TLR-5) during infection with wild-type *H. pylori* and isogenic *cag*PAI mutants. *H. pylori* triggered enhanced TLR-2 and TLR-5 expression in THP-1, HEK293-TLR2 and HEK293-TLR5 cells, but not in the HEK293 control. In addition, IL-8 and TNF-α cytokine secretion in THP-1 cells was induced in a *cag*PAI-dependent manner. Furthermore, we show that HEK293 cells are not competent for the uptake of T4SS-delivered CagA, and are therefore ideally suited for studying TLR signalling in the absence of T4SS functions. HEK293 control cells, which do not induce TLR-2 and TLR-5 expression during infection, only secreted cytokines in small amounts, in agreement with T4SS functions being absent. In contrast, HEK293-TLR2 and HEK293-TLR5 cells were highly competent for inducing the secretion of IL-8 and TNF-α cytokines in a *cag*PAI-independent manner, suggesting that the expression of TLR-2 or TLR-5 has profoundly changed the capability to trigger pro-inflammatory signalling upon infection. Using phospho-specific antibodies and luciferase reporter assays, we further demonstrate that *H. pylori* induces IRAK-1 and IκB phosphorylation in a TLR-dependent manner, and this was required for activation of transcription factor NF-κB. Finally, NF-κB activation in HEK293-TLR2 and HEK293-TLR5 cells was confirmed by expressing p65-GFP which was translocated from the cytoplasm into the nucleus. These data indicate that *H. pylori-*induced expression of TLR-2 and TLR-5 can qualitatively shift *cag*PAI-dependent to *cag*PAI-independent pro-inflammatory signalling pathways with possible impact on the outcome of *H. pylori-*associated diseases.

## Introduction

The innate immune system is evolutionarily conserved in higher eukaryotes and is the first line of defence for protecting hosts from invading microbial pathogens [1]–[3]. Toll-like receptors (TLRs) are surface-exposed pattern recognition receptors which can recognize molecular structures on pathogenic microbes associated molecular patterns (PAMPs). Bacterial molecules like lipopolysaccharides (LPS), lipoprotein, lipotheichoic acid, peptidoglycan, lipoarabinomannan (LAM), flagellin and CpG containing DNA are well-known examples of PAMPs [4]. TLRs recognize these compounds in the extracellular space and subsequently transduce signals through downstream effectors to mount innate immune responses against infections and pave way for successful adaptive immunity [3], [5]. Currently, eleven members of the TLR family have been identified in mammals. TLRs are type I integral membrane glycoproteins and on the basis of cytoplasmic homologous regions, they are included in the interleukin-1 receptor superfamily [6]. Two additional families of sensing receptors have also been discovered. Sensing of microorganisms intracellularly can be achieved by nucleotide oligomerization domain (NOD)-like receptors (NLRs) and Retinoic acid inducible gene-1 (RIG-1)-like receptors (RLRs). These two families comprise the intracellular sensors, of which NLRs recognize primarily molecules of bacterial origin while RLRs are involved in antiviral responses [7], [8]. Individual TLRs interact with different combinations of adapter proteins and activate various transcription factors such as nuclear factor (NF)-κB, activator protein-1 (AP-1) and interferon regulatory factors (IRF), driving a specific immune response [9]. TLRs trigger intracellular signalling pathways that result in the induction of inflammatory cytokines, type-I interferon (IFN) and chemokines. Microbial pattern recognition by TLRs in dendritic cells upregulate the expression of co-stimulatory molecules, which is essential for the initiation of adaptive immune responses in the host, thus linking innate and adaptive immunity [2], [10].

TLR receptor-ligand binding activates the interaction with MyD-88 (myeloid differentiation primary response protein-88) through the TIR domain in its cytoplasmic tail which in turn recruits IRAK-4 (Interleukin-1 receptor associated kinase-4) and thereby induces the association of another kinase member, IRAK-1 [11]. IRAK-1 is phosphorylated at threonine residue 209 (T-209), which results in a conformational change of the kinase domain and subsequent phosphorylation at threonine residue 387 (T-387) and other residues in the activation loop. This results in autophosphorylation and full enzymatic activity of IRAK-1. IRAK-1 hyperphosphorylation triggers its dissociation from MyD-88 without affecting the association with TRAF-6 [12]. Subsequently, phosphorylated IRAK-1 and TRAF-6 dissociate from the complex and bind to cell membrane protein TAB-1 (TAK-1 binding protein-1) followed by binding of TAK-1 (transforming growth factor-β-activated kinase) and TAB-2. Finally, IRAK-1 ubiquitinylation and degradation are rapidly induced and the remaining complex translocates into the cytoplasm. The latter complex associates with ubiquitin ligases such as UBC-13 (ubiquitin conjugating enzyme-13) and UEV-1a (ubiquitin conjugating enzyme E2 variant-1), leading to ubiquitinylation and degradation of TRAF-6 [13]. This activates TAK-1 and phosphorylation of the IKK (inhibitor of κB kinase) complex (IKKα, IKKβ and IKKγ) as well as MAP (mitogen activated protein) kinases. The IKK complex then phosphorylates IκB (inhibitor of κB) which leads to its ubiquitinylation and degradation. This ultimately releases NF-κB, which enables to translocate to the nucleus for transcription of pro-inflammatory genes [14], [15].

*Helicobacter pylori* is a major human pathogen colonizing the stomach of about half the world population and is the causative agent of chronic gastritis, gastric ulcer disease, and even gastric cancer [16]–[18]. Thus, the World Health Organization (WHO) classified *H. pylori* as a type-1 carcinogen. Colonization of the gastric epithelium by this bacterium and subsequent host/pathogen interactions lead to strong inflammatory changes in many cases and that paves way for the development of gastritis, ulceration and metaplasia [17]–[19]. Although the gastric epithelial cells represent a barrier against natural infections, immune cells are the real mediators of inflammation to ward off invading pathogens [20]. Infection of epithelial cells with *H. pylori* activates different molecular signalling mechanism including NF-κB, AP-1 and MAP kinases, leading to the expression and secretion of pro-inflammatory cytokines [21]. These mediators of inflammation include interleukins such as IL-1, IL-6, IL-8, IL-18 as well as tumour necrosis factor alpha (TNF-α) and others which change the microenvironment and even regulate deleteriously host cellular mechanisms at the site of infection [20]. The cytotoxin-associated genes pathogenicity island (*cag*PAI), a 40 kb stretch of DNA, encodes orthologs of components of a type IV secretion system (T4SS), and T4SS-positive *H. pylori* strains are associated with more severe disease [22], [23]. This T4SS forms a pilus, capable of injecting the CagA protein, peptidoglycan, and possibly other factors into host cells using integrin β1 as a receptor [24], [25]. Once delivered, CagA can be phosphorylated by tyrosine kinases Src and Abl and interacts with a large number of cellular proteins to trigger multiple effects on host signal transduction pathways to the nucleus, cytoskeleton and cell junctions [23]. Injected factors and structural components of the T4SS have been shown to influence membrane dynamics, actin-cytoskeletal rearrangements and the disruption of cell-to-cell junctions as well as proliferative, pro-inflammatory and anti-apoptotic nuclear responses in the host [17], [18], [22], [23].

The role of innate immune responses by sensing receptors in response to *H. pylori* infection is not yet completely understood and sometimes controverse in the literature. Numerous previous studies have reported the involvement of some TLRs and NOD proteins in the detection of *H. pylori*, and induction of pro-inflammatory and other responses [26]–[29]. For example, a landmark paper has shown that peptidogycan can be injected by the *H. pylori* T4SS to stimulate Nod1 and subsequently NF-κB [26], while other factors such as injected CagA may also play a role in stimulating NF-κB and IL-8 [30], [31]. However, it was also shown that *H. pylori* induces Nod1, which activates a RICK→TRAF3→TBK1→IKK→IRF7 pathway leading to the synthesis of type-I interferon, but not NF-κB [32]. In addition, there are numerous studies on the putative role of TLRs in *H. pylori* infection. Gastric epithelial cells are reported to be not sufficient to provide all the TLR molecules expressed on its surface for detection of *H. pylori*. Smith and co-workers reported that gastric epithelial cells recognize and respond to *H. pylori* infection at least in part through TLR-2 and TLR-5 [33]. TLR-4 mediated recognition of LPS from many bacteria is a key activator of the innate immune response in epithelial cells, while the purified form of *H. pylori* LPS is a relatively weak inducer. However, *H. pylori* LPS does activate NF-κB, but this was achieved via TLR-2 rather than TLR-4 [33]–[35]. In contrast to this, *H. pylori* LPS was reported to activate NF-κB in association with the expression of mitogen oxidase-1 (MOX-1), cyclooxygenase-II (COX II) and TNF-α transcripts in gastric pit cells, which express more TLR-4 but no TLR-2 [36]. Immunocytochemical studies using gastric mucosal biopsies have revealed that TLR-5 and TLR-9 expression on the gastric epithelium changed to an exclusive basolateral localization without detectable expression at the apical pole in *H. pylori* gastritis, however, TLR-4 expression was highly polarized in an apical and a basolateral compartment identical to the non-inflamed mucosa [37]. Interestingly, Mandell and co-workers reported that *H. pylori* LPS induced cytokine production was mediated through TLR-4, but the response to infecting bacteria such as *H. pylori*, *H. hepaticus* or *H. felis* were mediated through TLR-2 [38]. *H. pylori* infection of HEK-293 cells stably transfected with TLR-2 revealed many differently regulated genes as compared to HEK293 control cells, and eight of them showed changing expression patterns in infected epithelial cell lines [39]. Finally, it was also shown that chemically synthesized lipid-A (mimicking the natural lipid-A portion of LPS from *H. pylori*) has a low endotoxic potency and immunobiological activities, and is recognized by TLR-4 [40]. A study using recombinant FlaA protein and Δ*flaA* mutants of *H. pylori* revealed the less potent activity of TLR-5 mediated IL-8 secretion in epithelial cells [41]. A recent report attributed the FlaA evasion of TLR-5 is due to amino acids 89–96 of the N-terminal D1 domain and that may be responsible for low TLR-5 mediated activity on IL-8 secretion [42].

The above discussed studies indicate a highly complex scenario of possible involvement of TLRs in *H. pylori* infection, but this is still not fully understood. Our group is interested in the characterisation of *H. pylori* signalling in innate immune responses. Here we show that *H. pylori* infection of THP-1 monocytes induced not only the secretion of pro-inflammatory cytokines such as IL-8 and TNF-α in a *cag*PAI-dependent manner, but also upregulation of TLR-2 and TLR-5 expression. To investigate the role of TLR-2 and TLR-5 during infection in more detail and to clearly distinguish this from signalling induced by the *cag*PAI-dependent injection of peptidoglycan and CagA, we were screening for a cell model system in which T4SS effectors cannot be injected. We demonstrate that HEK293 is such useful system. Infected HEK293 wild-type cells show no *cag*PAI-dependent upregulation of cytokines and CagA cannot be injected and phosphorylated in these cells at all. By contrast, stable expression of TLR-2 or TLR-5 in HEK293 cells dramatically changes the capability of *H. pylori* to activate NF-κB, IL-8 and TNF-α as shown by luciferase reporter, ELISA and immunofluorescence assays. We also demonstrate that *H. pylori* infection of either TLR-expressing cell line induces the phosphorylation of IRAK-1 and IκB followed by NF-κB activation, which is in agreement with the hypothesis that upregulation of both TLR-2 and TLR-5 by *H. pylori* can switch *cag*PAI-dependent signalling to *cag*PAI-independent TLR signalling, thus may changing the outcome of infections substantially.

## Results and Discussion

### *H. pylori* infection of THP-1 cells upregulates TLR-2 and TLR-5 expression and cytokine secretion in a *cag*PAI-dependent manner

**Figure 1:**
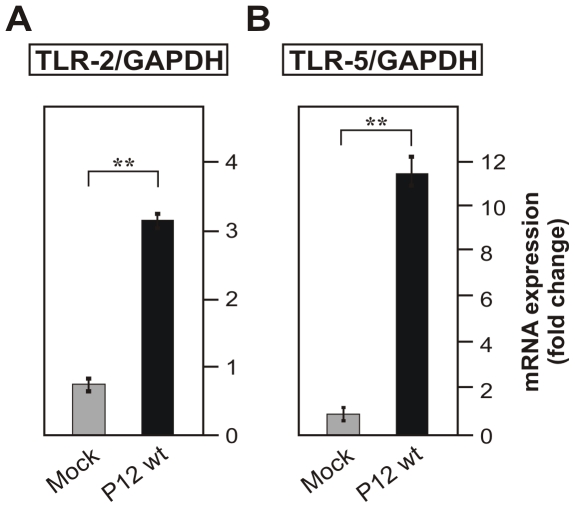
*H. pylori* infection of THP-1 cells induces TLR-2 and TLR-5 mRNA expression. The mRNA expression of TLRs was analyzed by Taqman Real Time PCR relative to the house keeping gene GAPDH using the 2−ΔΔ*C*t method [60]. Fold changes of mRNA expression of TLR-2 and TLR-5 in infected THP-1 cells (24 hours) were compared with that of uninfected mock control cells.

**Figure 2:**
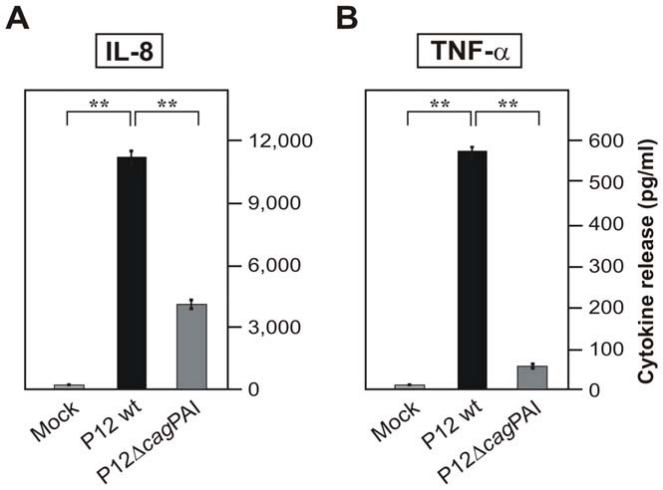
*H. pylori* infection of THP-1 cells upregulates the secretion of IL-8 and TNF-α in a *cag*PAI-dependent manner. Concentrations of IL-8 (A) and TNF-α (B) secreted from THP-1 cells after 24 hours of infection with wild-type *H. pylori* or an isogenic *cag*PAI mutant. TNF-α and IL-8 concentrations in the culture supernatants were quantified by ELISA.

TLR-2 and TLR-5 are two candidates of sensing receptors that maybe involved in interactions of host cells with *H. pylori* [33]–[35]. To investigate if the expression of both receptors can be regulated during infection, monocytic THP-1 cells were co-cultured with wild-type *H. pylori* for 24 hours followed by preparation of mRNA. The quantification of TLR-2 and TLR-5 mRNA expression was performed using Taqman Real Time PCR. As shown in Figure 1, *H. pylori* induced a more than 3- and 11-fold increase in TLR expression as compared to expression of the house keeping gene GAPDH, respectively. The protein expression of TLR-2 and TLR-5 in THP-1 cells in Western blots were found to be similarly up-regulated during infection with *H. pylori* (data not shown). We have then analyzed the secretion of IL-8 and TNF-α from THP-1 cells during infection with wild-type *H. pylori* and an isogenic Δ*cag*PAI deletion mutant by ELISA. The results indicate the induction of IL-8 and TNF-α secretion from THP-1 cells during infection with wild-type *H. pylori* (Figure 2). The induction of IL-8 and TNF-α, however, was not observed in infections with the Δ*cag*PAI mutant (Figure 2), which is in agreement with earlier studies indicating that T4SS-dependent activities trigger pro-inflammatory signalling [21].

### HEK293 cells are a useful system to study TLR-2 and TLR-5 signalling during *H. pylori* infection

**Figure 3:**
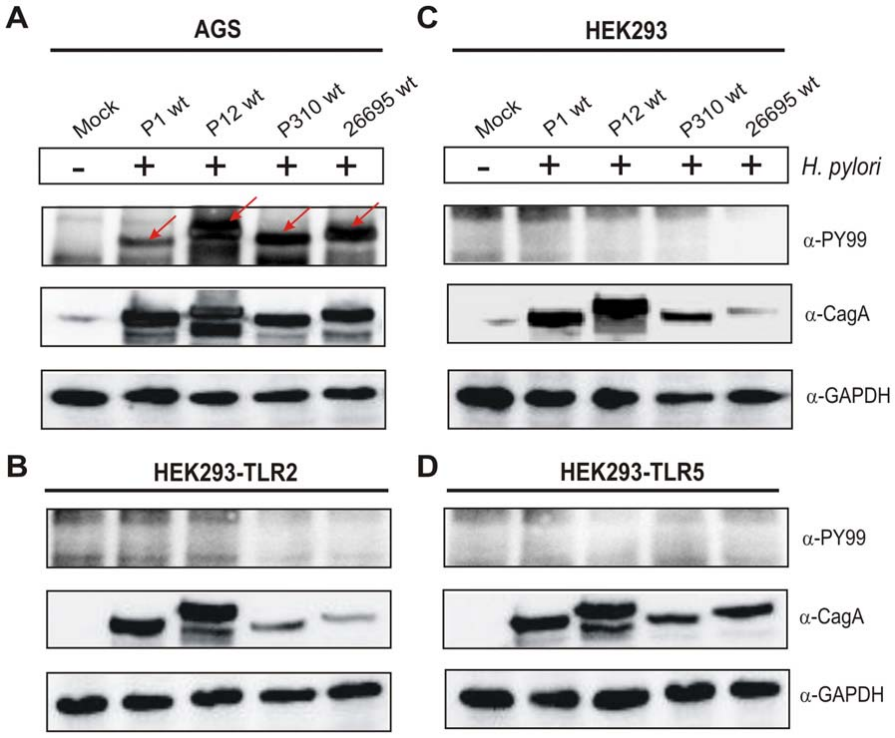
CagA injection by *H. pylori* cannot be achieved in infected HEK293 cell lines but in AGS gastric epithelial cells. Western blot analysis of (A) AGS, (B) HEK293-TLR2, (C) HEK293 and (D) HEK293-TLR5 cells infected with *H. pylori* wild-type strains P1, P12, P310 or 26695 for 6 hours. Phosphorylation of injected CagA was monitored using phosphotyrosine α-PY-99 and α-CagA antibodies. Red arrows indicate the position of phosphorylated CagA on the blot. Western blots for the house keeping gene GAPDH served as loading control.

To investigate the role of TLR-2 and TLR-5 in greater detail and to exclude signalling induced by the *cag*PAI-dependent injection of bacterial effectors, we next tested several cell lines for their susceptibility for injection and phosphorylation of CagA. Among the cell lines tested, CagA was strongly phosphorylated in infected AGS, MKN-28, MKN-45, KATO-III, THP-1, J774.A or HT-29 cell lines after 6 hours (Figure 3A, and data not shown), but not in wild-type HEK293 cells, or HEK293 cells stably transfected with expression constructs for human TLR-2 and TLR-5, respectively (Figure 3B–D). The experiments were performed under identical conditions. We also confirmed by phase microscopy the motility of each of the different bacterial strains and that they can bind to the cells with high efficiency (>80%), thus excluding the possibility that altered bacterial fitness or reduced host cell binding accounts for the observed CagA translocation defect. In addition, an initial centrifugation step (at 2,000 rpm for 10 minutes) of wild-type *H. pylori* onto HEK293 cells followed by 6 hours infection did not reveal any detectable signal for phosphorylated CagA (data not shown). Thus, the different HEK293 cell lines are not competent for the injection of CagA, and are therefore ideally suited for investigation of TLR signalling in the absence of T4SS functions.

### Role of *cag*PAI status on induction of TLRs and pro-inflammatory cytokines in HEK293-TLR2 and HEK293-TLR5 cells during *H. pylori* infection

**Figure 4:**
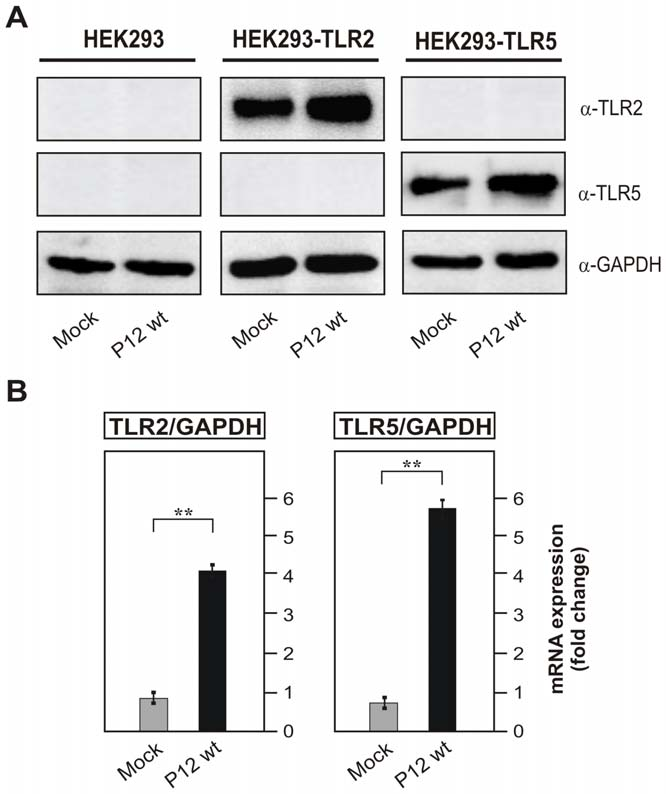
*H. pylori* infection induces enhanced expression of TLR-2 and TLR-5 proteins in HEK293-TLR2 and HEK293-TLR5 cells but not in HEK293 control cells. (A) Western blot analysis of TLR-2 and TLR-5 proteins in HEK293, HEK293-TLR2 and HEK293-TLR5 cell lines. (B) Fold changes of mRNA expression of TLR-2 and TLR-5 in HEK293, HEK293-TLR2 and HEK293-TLR5 cell lines infected with *H. pylori* as compared to uninfected mock control cells. The mRNA expression of TLRs was analyzed by Taqman Real Time PCR relative to the house keeping gene GAPDH using the 2−ΔΔ*C*t method [60].

**Figure 5:**
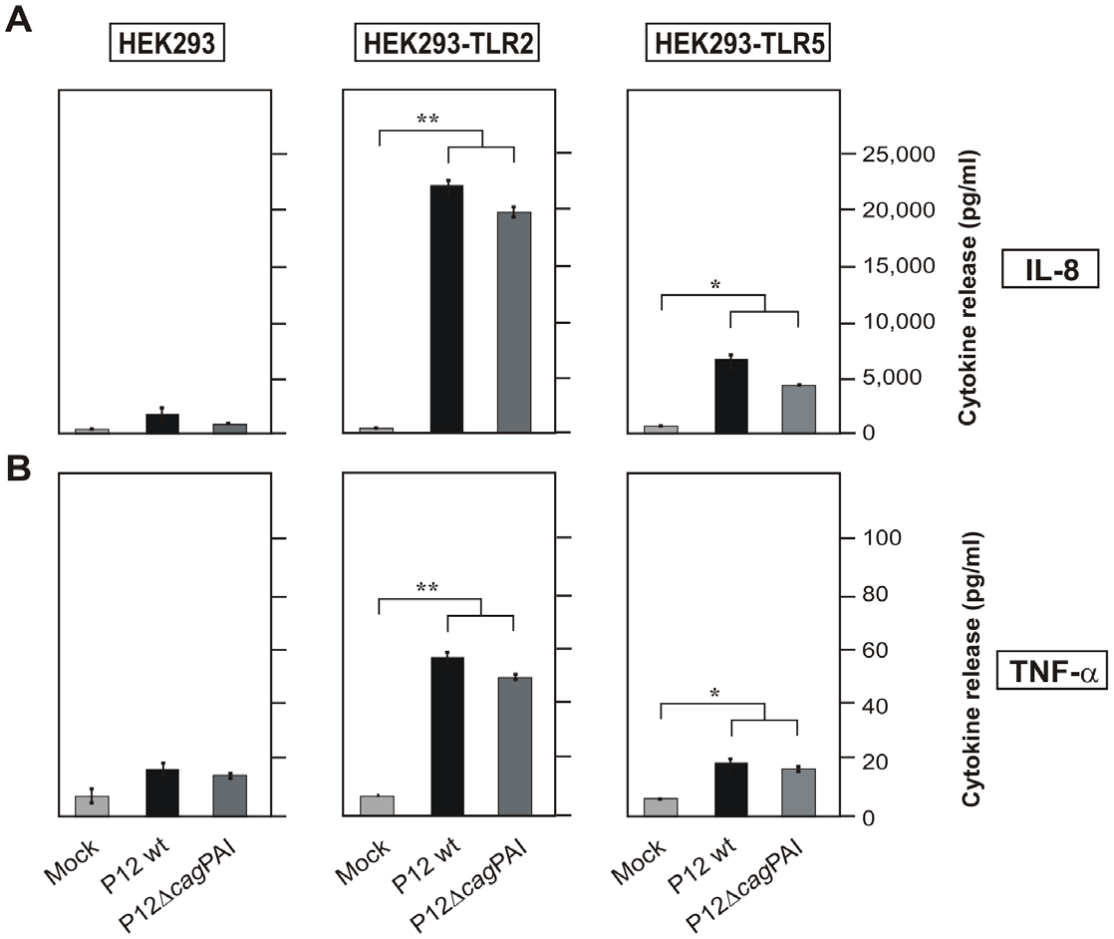
IL-8 and TNF-α secretion in HEK293 cell lines during *H. pylori* infection is mediated in a *cag*PAI-independent fashion. Concentrations of IL-8 (A) and TNF-α (B) secreted from HEK293, HEK293-TLR2 and HEK293-TLR5 cell lines after 24 hours of infection with wild-type *H. pylori* and an isogenic Δ*cag*PAI mutant. TNF-α and IL-8 concentrations in the culture supernatants were analyzed by ELISA.

To further confirm the usefulness of the HEK system, we have infected HEK293 wild-type, HEK293-TLR2 and HEK293-TLR5 cells with *H. pylori* for 8 hours followed by protein preparation for Western blotting or mRNA isolation for Taqman Real Time PCR. We found significant up-regulation of TLR-2 in HEK293-TLR2 cells and TLR-5 in HEK293-TLR5 cells both at protein level (Figure 4A) and mRNA level (Figure 4B), which was similar to the elevated TLR expression pattern observed in infected THP-1 cells (Figure 1). In addition, we performed cytokine ELISA's of cell supernatants for secreted IL-8 and TNF-α. We only found very small amounts of induced IL-8 or TNF-α in supernatants of infected HEK293 control cells, which is in agreement with the observation that T4SS functions are absent (Figure 5A, B). In contrast, infection of HEK293-TLR2 and HEK293-TLR5 cell lines with wild-type *H. pylori* strongly induced the secretion of a few thousand-fold IL-8 and several-fold TNF-α (Figure 5A, B). Interestingly, a parallel infection experiment of HEK293-TLR2 and HEK293-TLR5 cell lines with the isogenic Δ*cag*PAI deletion mutant also induced very high amounts of IL-8 and TNF-α similar to wild-type bacteria (Figure 5A, B), suggesting that the expression of either TLR-2 or TLR-5 has changed the capability of HEK293 cells to trigger *H. pylori*-induced pro-inflammatory signalling in a *cag*PAI-independent fashion.

### *H. pylori* induces IRAK-1 and IκB phosphorylation during infection with HEK293-TLR2 and HEK293-TLR5 cell lines but not in HEK293 wild-type cells

**Figure 6:**
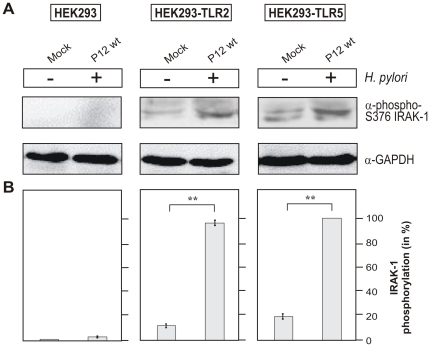
*H. pylori* infection of different HEK293 cell lines induces IRAK-1 phosphorylation at Ser-376 in a TLR-2- or TLR-5-dependent fashion. (A) Western blot analysis of IRAK1 phosphorylation in HEK293, HEK293-TLR2, and HEK293-TLR5 after 2 hours of infection. Blots for house keeping gene GAPDH were used as loading control. (B) Densitometric measurement of band intensities revealed the percentage of IRAK-1 phosphorylation per sample. The strongest band was set 100% as indicated.

**Figure 7:**
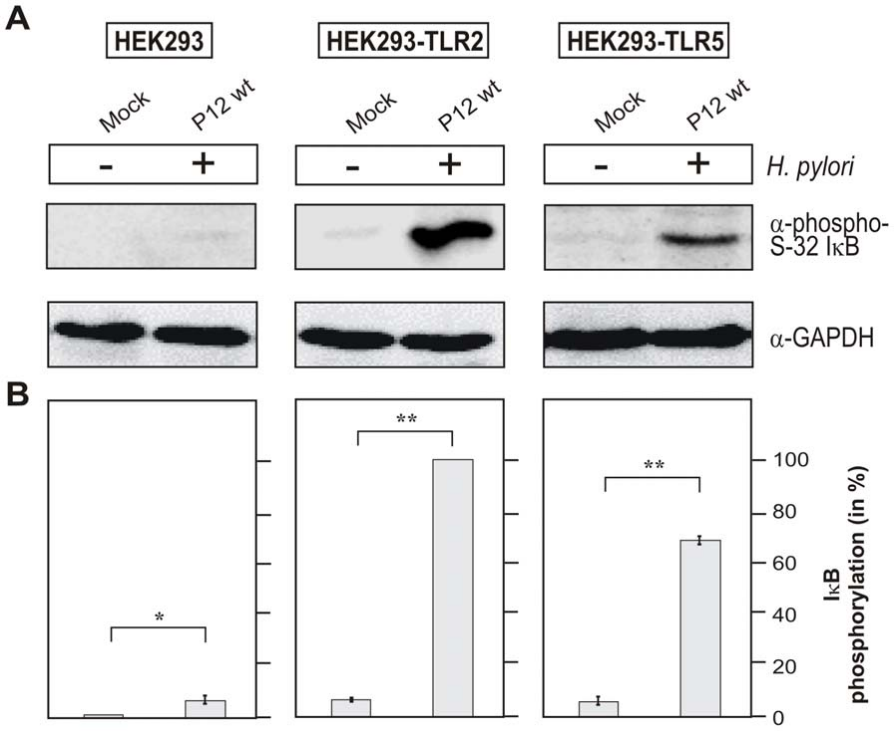
*H. pylori* infection of different HEK293 cell lines induces IκB phosphorylation at Ser-32 in a TLR2- or TLR-5-dependent fashion. (A) Western blot analysis of IκB phosphorylation in HEK293, HEK293-TLR2, and HEK293-TLR5 after 2 hours of infection. Blots for house keeping gene GAPDH were used as loading control. (B) Densitometric measurement of band intensities revealed the percentage of IκB phosphorylation per sample. The strongest band was set 100% as indicated.

To investigate the functional status of TLR-2 and TLR-5 in HEK293 cells, we next analyzed the initiation of classical TLR signalling through IRAK-1 phosphorylation. IRAK-1 is commonly phosphorylated at several threonine and serine residues which are involved in its activation as described above. Activation-specific phospho antibodies are available for the phosphorylated serine residue 376 (S-376) in IRAK-1. For this purpose, HEK293 wild-type, HEK293-TLR2 and HEK293-TLR5 cells were infected with *H. pylori* for 2 hours followed by Western blotting using the α-phospho S-376 IRAK-1 antibody. We found that *H. pylori* induced IRAK-1 phosphorylation at S-376 in HEK293-TLR2 and HEK293-TLR5 cell lines but not in the wild-type HEK293 control cells (Figure 6A, B). Furthermore, the same cell lysates as prepared above were probed for IκB phosphorylation as also indicative for the activated NF-κB pathway. The results show that *H. pylori* induces the phosphorylation of IκB at serine residue 32 (S-32) in HEK293-TLR2 and HEK293-TLR5 cell lines but not in the wild-type HEK293 control cells (Figure 7A, B). These findings suggest that *H. pylori* are able to induce both activation of IRAK-1 and IκB in a TLR-dependent manner as required for the onset of transcription factor NF-κB.

### NF-κB and AP-1 activation in HEK293-TLR2, HEK293-TLR5 and HEK293 wild-type cells during infection with *H. pylori*

**Figure 8:**
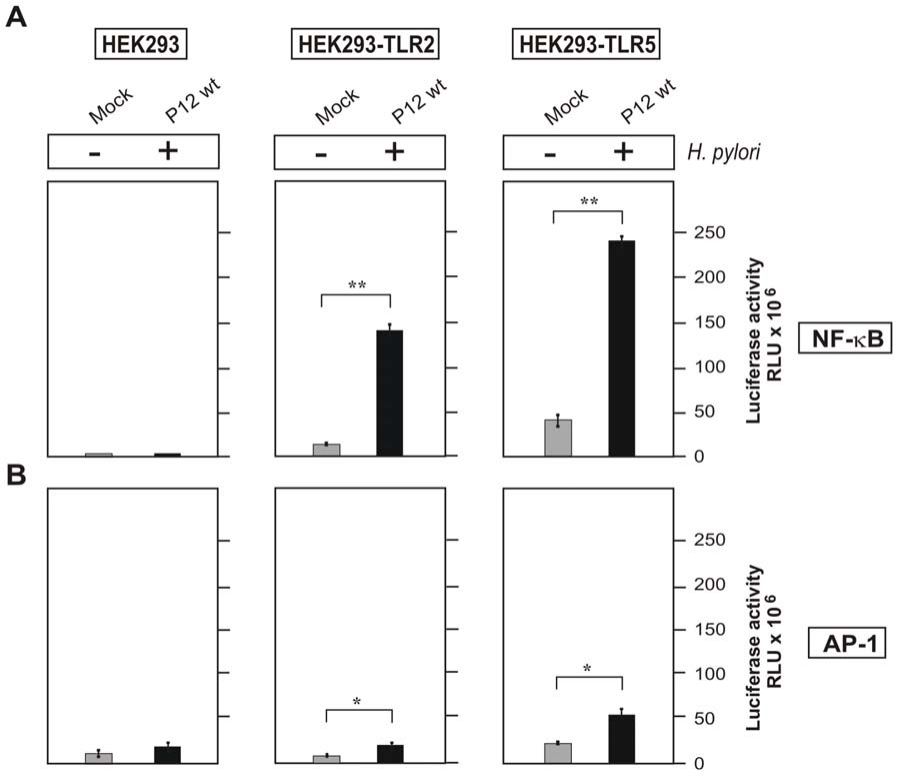
*H. pylori* infection of different HEK293 cell lines induces NF-κB and AP-1 activation in a TLR2- or TLR-5-dependent fashion. NF-κB and AP-1 luciferase reporter constructs were transfected into HEK293, HEK293-TLR2, and HEK293-TLR5 cells for 48 hours and followed by infection with *H. pylori* for 5 hours. The NF-κB and AP-1 luciferase reporter expression was analyzed as function of activation.

**Figure 9:**
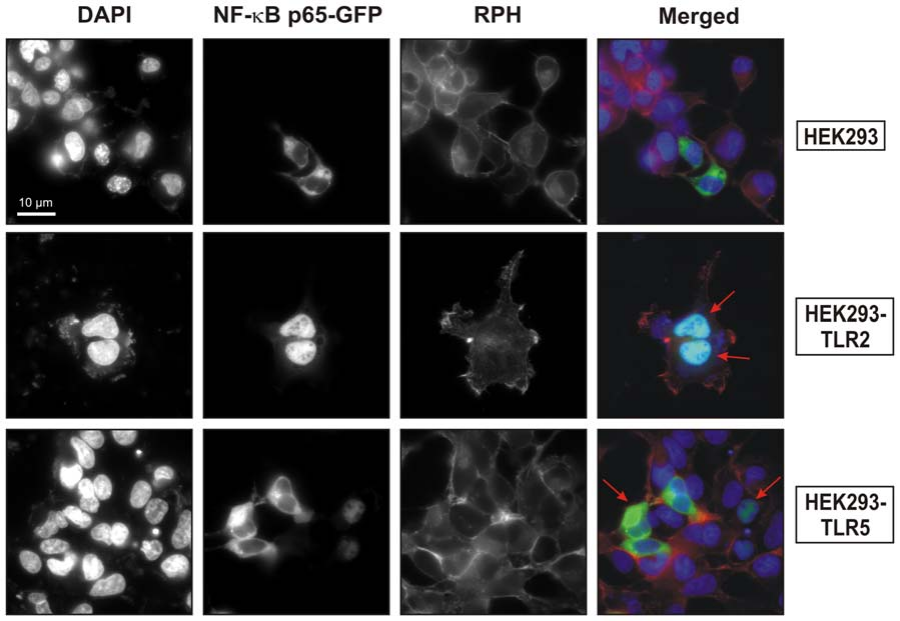
Activation of NF-κB-GFP in HEK293 cell lines is dependent on TLR-2 and TLR-5. Immunofluorescence of HEK293 cell lines transfected with NF-κB-p65 subunit (p65-GFP, green) for 48 hours followed by infection with *H. pylori* for 3 hours. Infection of HEK293-TLR2 and HEK293-TLR5 cells with *H. pylori* showed a translocation of p65-GFP into the nucleus (arrows), whereas infection of HEK293 control cells did not induce a nuclear translocation of p65-GFP. Rhodamine-phalloidine (RPH, red) was used to visualize filamentous actin in the cells and DAPI (blue) to visualize the nucleus and bacteria.

As next, NF-κB activity was studied using a luciferase reporter system with the respective AP-1 construct as control. To this end, the different HEK293 cell lines were transiently transfected with NF-κB luciferase and AP-1 luciferase gene constructs for 48 hours, followed by infection with *H. pylori* for 5 hours. Cell lysates were then prepared and the activation of both transcription factors was quantified by luciferase activity counting. NF-κB or AP-1 luciferase activities were very small both in non-infected and infected HEK293 wild-type control cells (Figure 8A, B). In contrast, NF-κB luciferase activity was strongly elevated in HEK293-TLR2 and HEK293-TLR5 cells infected with *H. pylori* (Figure 8A). Interestingly, AP-1 luciferase activity was only moderately induced in infected HEK293-TLR2 and HEK293-TLR5 cells, and was much lower as compared to NF-κB activity (Figure 8A, B), Finally, we confirmed NF-κB activation in HEK293-TLR2 and HEK293-TLR5 cells using a transfected NF-κB p65-GFP construct. As shown in Figure 9, *H. pylori* infection of the HEK293-TLR2 and HEK293-TLR5 cells showed translocation of p65-GFP from the cytoplasm into the nucleus (arrows).

### Conclusion

Mounting evidence supports the view that innate immune responses to microbes participate in the development of severe gastrointestinal disorders. With the characterization of the innate immune system, we have begun to understand the adaptations that intestine has created to the colonising microbiota. The interaction between the microbiota and the intestinal mucosa through TLRs is required to maintain intestinal homeostasis. In particular, intestinal epithelial cells and lamina propria immune cells must respond to breaches in the mucosal barrier by activating TLR-dependent signalling pathways that trigger increased epithelial proliferation, wound healing and recruitment of acute inflammatory cells. In the setting of chronic inflammation such as *H. pylori* infection in the stomach, the process of TLR interaction and activation is relatively little understood. However, recent reports showed that TLR-2, TLR-9 (recognising *H. pylori* DNA) and RIG-I (recognising *H. pylori* RNA) through the interaction with respective ligands and downstream signalling were able to activate dendritic cells leading to adaptive immune responses against *H. pylori* infection [27]–[29]. Highly purified *H. pylori* LPS preparations significantly induced pro-inflammatory reactions via the receptor complex containing TLR-2/TLR-1 or TLR-2/TLR-6 but not TLR-4 [43]. In contrast, a study published very recently showed that *H. pylori* LPS markedly enhanced IL-8 production induced by *E. coli* LPS through upregulating TLR-4 via TLR-2 and the MEK1/2-ERK1/2 MAP kinase pathway and augmented the proliferation rate of gastric epithelial cells. This activation was mediated through LPS carrying a weakly antigenic epitope, which is frequently found in gastric cancers, than by LPS carrying a highly antigenic epitope, which is associated with chronic gastritis [44]. On the other hand, the *H. pylori* factor activating TLR-5 is not flagellin and therefore still unknown [41], [42].

In the present report we show that *H. pylori* infection of THP-1 monocytes induced the secretion of IL-8 and TNF-α in a *cag*PAI-dependent manner, which was associated with enhanced TLR-2 and TLR-5 expression. We investigated the function of TLR-2 and TLR-5 during infection in more detail. We demonstrate that HEK293 is a very useful cell system in which T4SS effectors cannot be injected and used it to investigate *H. pylori*-induced TLR signalling in the absence of *cag*PAI-dependent signalling effects. We could show that expression of TLR-2 or TLR-5 in HEK293 cells dramatically changes the capability of *H. pylori* to activate NF-κB, IL-8 and TNF-α as demonstrated in different molecular assays. The *H. pylori*-induced signalling downstream of TLR-2 and TLR-5 involves the activation of IRAK-1 and IκB phosphorylation followed by NF-κB stimulation and cytokine release. Thus, upregulation of both TLR-2 and TLR-5 during the course of *H. pylori* infection can switch *cag*PAI-dependent signalling to *cag*PAI-independent TLR signalling, which may impact disease development. Our findings are in part supported by an earlier study which showed that IL-8 secretion induced by *H. pylori* from HEK293 cells has been augmented by the expression of TLR-2 or TLR-5 and that coincided with increased p38 activation and phosphorylation of the transcription factor ATF2 [45]. In addition, we have been able to show that *H. pylori* induced NF-κB stimulation and release of IL-8 and TNF-α in a *cag*PAI-dependent way in THP-1 monocytes and the expression of TLR-2 and TLR-5, as studied in HEK293 cells, support a model for a switch to *cag*PAI-independent signalling at late time points of infection.

The switching of *cag*PAI-dependent to *cag*PAI-independent signalling by induction of TLR-2 and TLR-5 *in vitro* may have important implications on *H. pylori-*associated pathologies *in vivo*. For example, in mixed infections of *cag*PAI-negative and *cag*PAI-positive strains the signalling can be severely influenced and augmented in a mutually supportive way and turn the status to pro-inflammatory. Interestingly, in biopsies from *H. pylori-*positive gastritis patients, expression of TLR-5 and TLR-9 in the gastric epithelium changed to an exclusive basolateral localization without detectable expression at the apical pole [37]. This is an interesting observation because the receptor for the *H. pylori* T4SS is integrin β1 and also localised to the basolateral side of the epithelium [24]. This suggests that several *H. pylori* receptors are found both on the apical and basolateral sides of gastric epithelial cells and there maybe even crosstalk between some of them [46]. However, the increased production of TNF-α through TLR-2 and TLR-5 signalling might be a reason for inducing apoptosis of gastric cells at the colonized area of the stomach. The apoptotic bodies can also be a source of endogenous ligands for TLR molecules, which (like TLR-7 and TLR-9) have been reported for the high proportion of auto-antibodies binding DNA, RNA or macromolecular complexes that contain DNA or RNA, and that are commonly associated with systemic autoimmune diseases such as systemic lupus erythematosus (SLE), scleroderma and Sjögren's syndrome. *H. pylori* has been found to be associated with a number of autoimmune disorders, such as rheumatoid arthritis, autoimmune thyroiditis, Sjögren's syndrome, Schonlein-Henoch purpura, and autoimmune thrombocytic purpura [47]. The ability of microbial TLR ligands to trigger disease onset in experimental models of arthritis, multiple sclerosis, experimental allergic encephalomyelitis, myocarditis, diabetes and atherosclerosis have also been reported. Endogenous ligands for TLR-2 and TLR-4 such as hyaluronate, heparan sulphate, fibronectin and heat shock proteins have also been implicated in the pathogenesis of autoimmune diseases such as rheumatoid arthritis [48]. *H. pylori* is also reported to provide epitopes cross-reactive to H+, K+-ATPase and this may lead to the expansion of cross-reactive and auto-reactive T cells and T cell-dependent B cell activation, which can be attributed to the parietal cell loss due to autoimmune gastritis. Autoreactive T cells produce high concentration of TNF-α and IFN-γ, which can in turn increase the MHC class II and co-stimulatory molecule expression in gastric epithelial cells and favouring the presentation of peptides by such non-professional antigen-presenting cells [49]. A very recent study using mouse models of infection with *Helicobacter felis* (a close relative of *H. pylori*) showed that B cells activated by *Helicobacter* TLR-2 ligands induce IL-10-producing CD4(+)CD25(+) T regulatory-1 (Tr-1)-like cells and these B cell-induced Tr-1 cells acquire suppressive activities *in vitro* and suppress excessive gastric *Helicobacter*-associated immunopathology *in vivo* [50]. These facts along with the ability of *H. pylori* to switch signals into a TLR-dependent pathway is showing that it may influence under certain circumstances not only gastric pathologies but also a range of other complex responses.

## Materials and Methods

### Eukaryotic cell culture

The monocytic leukaemia cell line THP-1 (ATCC TIB 202) was cultured in RPMI-1640 medium supplemented with 10% (vol/vol) heat-inactivated fetal bovine serum (FBS) (Invitrogen, Germany) 1% antibiotic and antimycotic solution (Sigma-Aldrich, Germany). The transformed human embryonic kidney cells (HEK293 wild-type, ATCC CRL-1573) were cultured in Dulbecco's Modified Eagle Medium (DMEM) containing 4.5 g/L D-glucose, 4 mM L-glutamine, 110 mg/L sodium pyruvate,10% FBS (Invitrogen, Germany) and was supplemented with 1% antibiotic and antimycotic solution (Sigma-Aldrich, Germany). HEK293 cells stably transfected with pUNOhTLR2 and pUNOhTLR5 constructs (Invivogen, France), respectively, were cultured in DMEM containing 4.5 g/L D-glucose, 4 mM L-glutamine, 110 mg/L sodium pyruvate, 10% FBS (Invitrogen, Germany) and was supplemented with 1% antibiotic and antimycotic solution (Sigma, Germany) and 10 µg/ml blasticidin (Invivogen, France). Cells were maintained in 75-cm^2^ tissue culture flasks (Greiner-Bio-One, Germany) at 37°C in incubators with 5% CO_2_. All other cell lines (AGS, MKN-28, MKN-45, KATO-III, J774.A or HT-29) were cultured as described [51]. Prior to bacterial infection, all cells were incubated in antibiotic-free medium overnight.

### *H. pylori* growth and conditions for infection

The *H. pylori* strains employed in this study include the wild-type strains. P1, P310, 26695, P12 and the isogenic mutant of P12Δ*cag*PAI in which the entire *cag*PAI was deleted [52]–[54]. All *H. pylori* strains were grown in thin layers on horse serum agar plates supplemented with vancomycin (10 mg ml−1), nystatin (1 mg ml−1) and trimethoprim (5 mg ml−1), and in case of the mutants with kanamycin (8 mg ml−1) or chloramphenicol (4 mg ml−1), respectively. All antibiotics were obtained from Sigma. Incubation of the bacteria was performed at 37°C for 2 days in an anaerobic jar containing a campygen gas mix of 5% O_2_, 10% CO_2_ and 85% N_2_ (Oxoid, Germany). *H. pylori* grown on agar plates was harvested and resuspended in Phosphate Buffered Saline (PBS, pH 7.4) using a sterile cotton swab (Raucotupf, Lohmann & Rascher, Germany). The bacterial concentration was measured as optical density (OD) at 550 nm using an Eppendorf spectrophotometer. This was also cross checked with colony-forming units (CFU) grown on horse serum agar plates after serial dilution of the bacterial suspension. The eukaryotic cells grown in medium without antibiotics and antimycotics were infected with *H. pylori* at a multiplicity of infection (MOI) of 50. The uninfected cells were incubated with equal amount of PBS as control.

### RNA extraction, cDNA preparation and Taqman Real Time PCR

THP-1 cells and HEK293 cell lines infected with *H. pylori* were used for the isolation of mRNA. The THP-1 cells infected with *H. pylori* for the required time periods were separated from bacteria by centrifugation at 150×*g* for 5 minutes at 4°C. The supernatant containing the majority of bacteria were removed carefully and the cell pellet was washed with PBS before adding lysis buffer of the RNeasy Mini Kit (Qiagen, Germany). In case of HEK293 cell lines infected with *H. pylori*, the supernatant was carefully removed and the monolayer was washed with PBS before adding 300 µl of lysis buffer directly to the cells. Then, mRNA has been extracted by the protocol provided by the manufacturer (Qiagen). The purified mRNAs from *H. pylori* infected cell lines were used to prepare cDNA by RT-PCR. The RT-PCR in principle synthesizing the first strand complimentary DNA from mRNA with the help of Oligo dT primers, which bind to Poly A tail of all eukaryotic mRNAs, and Moloney Murine Leukaemia Virus (M-MLV) Reverse Transcriptase (Invitrogen, Germany). The Taqman Real Time PCR uses the fluorescent oligonucleotide probes labeled with one reporter dye at 5′ end of the probe and quencher dye at the 3′ end along with the usual sense and anti-sense primers used in PCR [55]. These probes are designed to hybridize to an internal region of a PCR product. The probe when attached to the template or unattached, the quencher dye reduces the fluorescence from reporter dye by the mechanism of Fluorescence Resonance Energy Transfer (FRET), which is the inhibition of one dye caused by another without emission of a proton. When the Taqman PCR proceeds, the probe annealed to the template will be removed by the exonuclease activity of Taq polymerase and this separates the reporter dye from the quencher and that increases the emission of fluorescence. DNase treated mRNA samples isolated from *H. pylori*-infected cells were used for first strand cDNA preparation using reverse transcriptase. Taqman probes and primers prepared using Primer express software and Taqman gene expression assay kits (Applied Biosystems, USA) were used for the analysis.

### Quantification of cytokines by immunoassay

THP-1 and HEK293 cell lines were incubated for 24 hours with *H. pylori*, and PBS-incubated control cells served as a negative control. The culture supernatants were collected and stored at −80°C until assayed. IL-8 and TNF-α concentration in the supernatant were determined by standard ELISA with commercially available assay kits according to manufacturer's procedures (Becton Dickinson, Germany).

### SDS-PAGE and immunoblot analysis

Infected and control cells were harvested and mixed with equal amounts of 2×SDS-PAGE buffer and boiled for 5 minutes. Proteins were separated by SDS-PAGE on 6–12% polyacrylamide gels and blotted onto PVDF membranes (Immobilon-P, Millipore, USA) as described [56], [57]. Before addition of the antibodies, membranes were blocked in TBS-T (140 mM NaCl, 25 mM Tris-HCl pH 7.4, 0.1% Tween-20) with 3% BSA or 5% skim milk for 1 hour at room temperature. Phosphorylated and non-phosphorylated CagA proteins were detected by incubation of the membranes with a mouse monoclonal α-phosphotyrosine antibody PY99 (Santa Cruz, USA) and a rabbit polyclonal α-CagA antibody (Austral Biologicals, USA). Monoclonal antibody recognizing phosphorylated IκB at S-32 and rabbit polyclonal antibody recognizing phoshorylated IRAK-1 at S-376 were purchased from NEB cell signalling (USA). TLR-2, TLR-5 and GAPDH expression was monitored using antibodies from Santa Cruz (USA). As secondary antibodies, horseradish peroxidase-conjugated antimouse or α-rabbit polyvalent rabbit and pig immunoglobulin, respectively, were used (Dako, Germany). Antibody detection was performed with the ECL Plus chemoluminescence Western Blot system for immunostaining (Amersham Pharmacia Biotech, Germany).

### Transient transfection of HEK293 cells

HEK-cells were transfected with 4 µg of the NF-κB p65-GFP [58] or NF-κB luciferase or AP-1 luciferase constructs [59] for 48 hours with TurboFect reagent according to the manufacturer's instructions (Fermentas, Germany).

### Immunofluorescence analysis

Transfected cells were infected with *H. pylori* for 3 hours. Cells were fixed with 3.8% PFA and stained with rhodamine-phalloidine to visualize the actin cytoskeleton of the cells and DAPI to visualize the nucleus. Samples were analyzed using the fluorescence microscope (Leica DMRE7, Leica, Germany) equipped with a CCD camera (Spot RT, Diagnostic Instruments, Burroughs, MI, USA) and a 63/1.4 objective. Separate images were taken in the corresponding channels, and later merged using ImageJ™ software (NIH, USA).

### Quantification of NF-κB or AP-1 activity by luciferase reporter assay

Transfected cells were infected with *H. pylori* for 5 hours and analyzed by luciferase assay using the Dual-Luciferase Reporter Assay System according the manufactures instruction (Promega, USA). Briefly, cells were harvested by passive lysis, the protein concentration was measured with Precision Red (Cytoskeleton, USA) and the lysates were equalized by adding passive lysis buffer. The luciferase activity was measured by using a Plate Luminometer (MITHRAS LB940 from Berthold, Germany).

### Statistical Analysis

All experiments were done at least three times with similar results. The data were evaluated using Student t-test with SigmaStat statistical software (version 2.0). *P* values = p<0.05 (*) and p<0.005 (**) were considered as statistically significant.
