# Correction: Induction of TLR-2 and TLR-5 Expression by *Helicobacter pylori* Switches *cag*PAI-Dependent Signalling Leading to the Secretion of IL-8 and TNF-α

**DOI:** 10.1371/journal.pone.0141721

**Published:** 2015-10-23

**Authors:** Suneesh Kumar Pachathundikandi, Sabine Brandt, Joseph Madassery, Steffen Backert

In Panel A of Fig 6, the blot images for the GAPDH lanes in the HEK293-TLR2 and HEK293-TLR5 panels were erroneously duplicated. Please see Figs 10 and 11 to view the raw blots for the GAPDH lanes.

**Fig 10:**
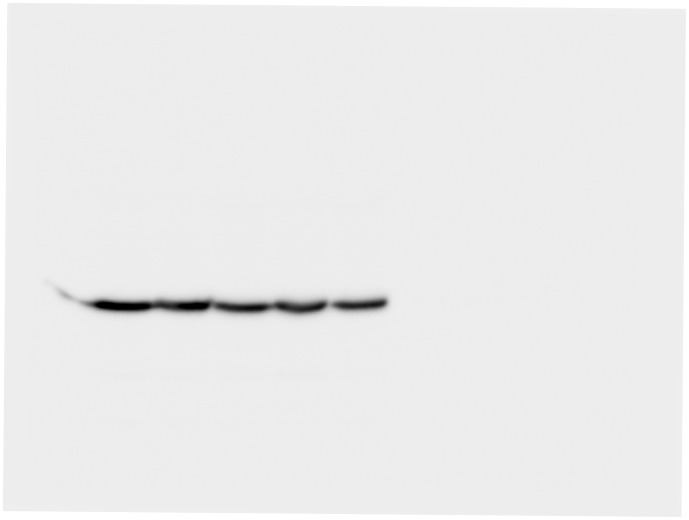
Raw blot for HEK293-TLR2 GAPDH

**Fig 11:**
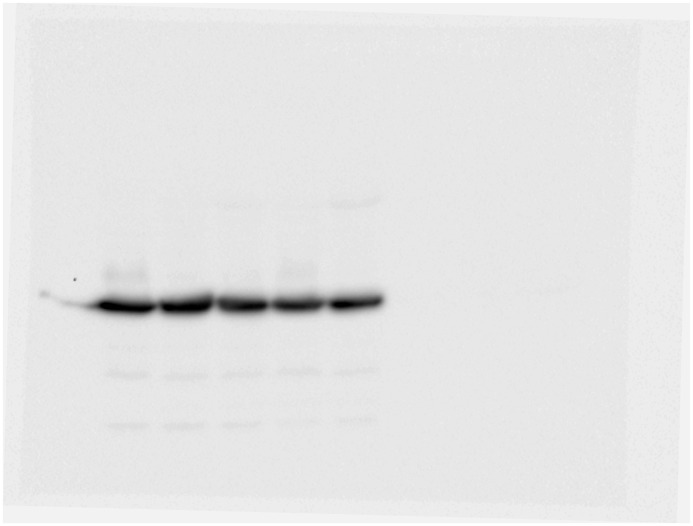
Raw blot for HEK293-TLR5 GAPDH
