# Correction: Correction: Induction of TLR-2 and TLR-5 Expression by *Helicobacter pylori* Switches *cag*PAI-Dependent Signalling Leading to the Secretion of IL-8 and TNF-α

**DOI:** 10.1371/journal.pone.0144365

**Published:** 2015-12-02

**Authors:** The PLOS ONE Staff

There is an error in the Correction published on October 23, 2015. The correction should refer to Panels B and D of Fig 3, not Panel A of Fig 6. The publisher apologizes for the error. The correct text is:

**Fig 3:**
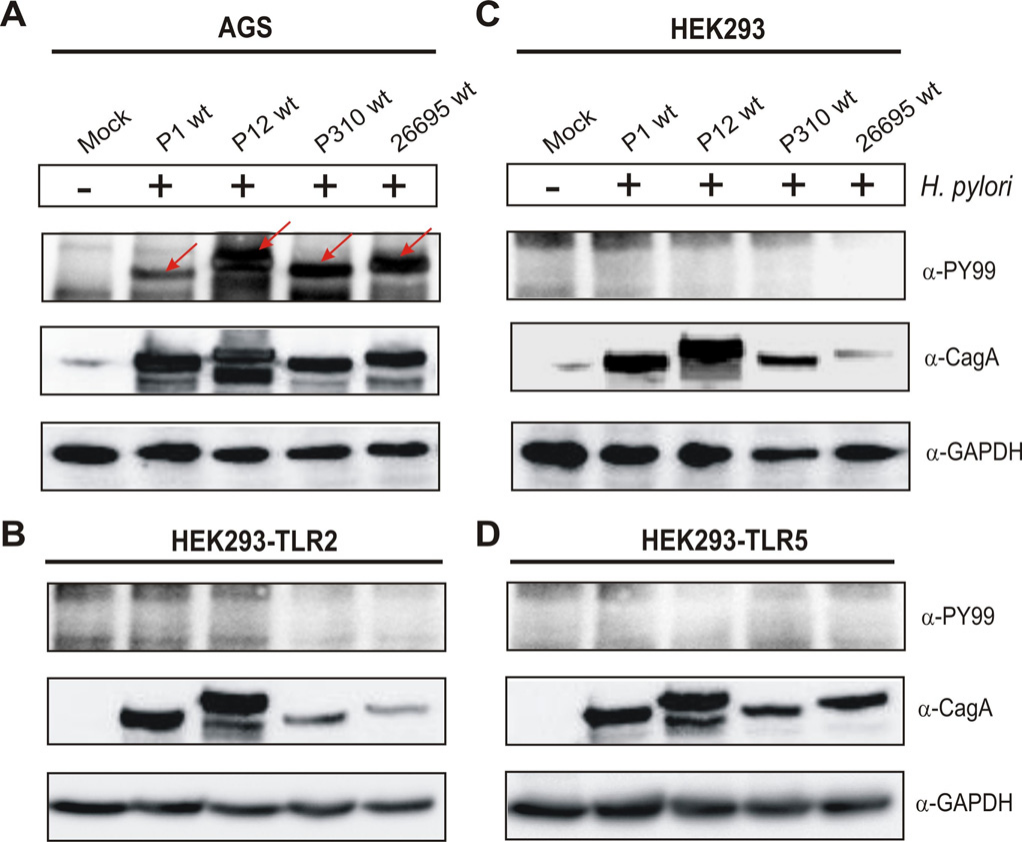
CagA injection by H. pylori cannot be achieved in infected HEK293 cell lines but in AGS gastric epithelial cells. Western blot analysis of (A) AGS, (B) HEK293-TLR2, (C) HEK293 and (D) HEK293-TLR5 cells infected with H. pylori wild-type strains P1, P12, P310 or 26695 for 6 hours. Phosphorylation of injected CagA was monitored using phosphotyrosine α-PY-99 and α-CagA antibodies. Red arrows indicate the position of phosphorylated CagA on the blot. Western blots for the house keeping gene GAPDH served as loading control.

In Panels B and D of Fig 3, the blot images for the GAPDH lanes in the HEK293-TLR2 and HEK293-TLR5 panels were erroneously duplicated. Please see Figs 10 and 11 to view the raw blots for the GAPDH lanes.

**Fig 10:**
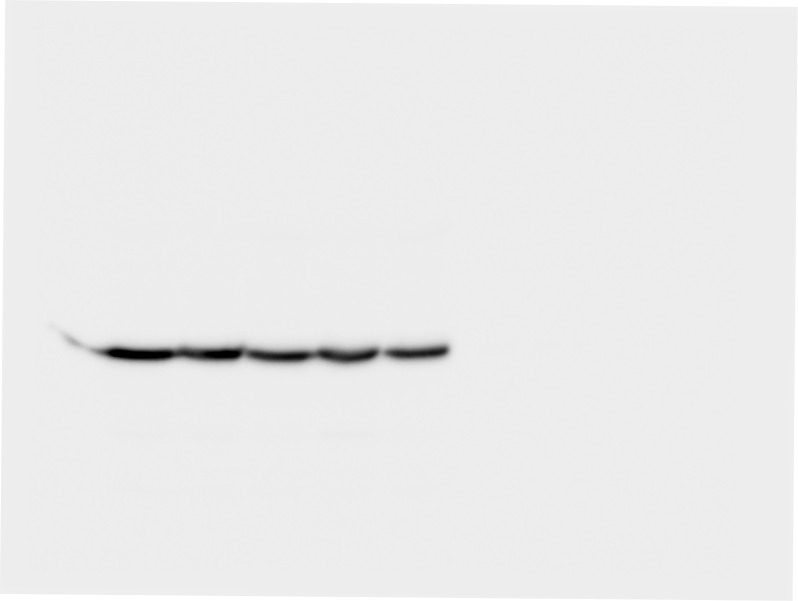
Raw blot for HEK293-TLR2 GAPDH

**Fig 11:**
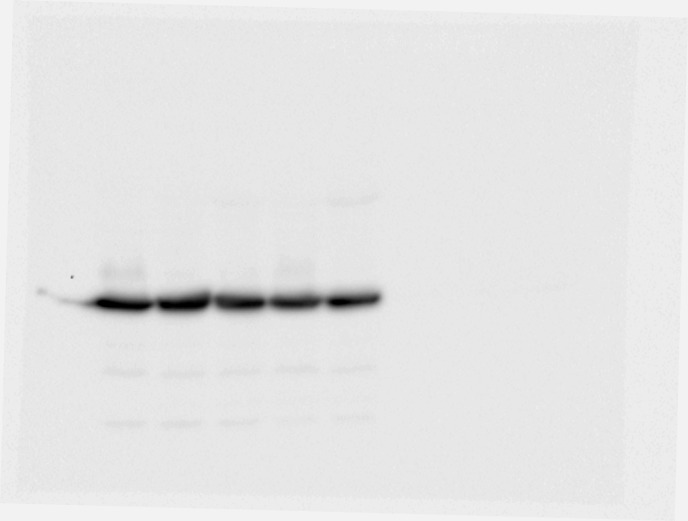
Raw blot for HEK293-TLR5 GAPDH
